# Relationship Between the Practice of Physical Activity and Physical Fitness in Physical Education Students: The Integrated Regulation As a Mediating Variable

**DOI:** 10.3389/fpsyg.2020.01910

**Published:** 2020-07-29

**Authors:** Gemma María Gea-García, Noelia González-Gálvez, Alejandro Espeso-García, Pablo J. Marcos-Pardo, Francisco Tomás González-Fernández, Luis Manuel Martínez-Aranda

**Affiliations:** ^1^Faculty of Sports, San Antonio Catholic University (UCAM), Murcia, Spain; ^2^Health, Physical Activity, Fitness and Motor Control Performance Research Group (GISAFFCOM), San Antonio Catholic University (UCAM), Murcia, Spain; ^3^Department of Physical Activity and Sport Sciences, CESAG, Comillas Pontifical University, Madrid, Spain; ^4^Neuroscience of Human Movement Research Group, San Antonio Catholic University (UCAM), Murcia, Spain

**Keywords:** physical activity, physical fitness, integrated regulation, IPAQ, PLOC-2, schoolchildren

## Abstract

The practice of physical activity (PA) contributes to the prevention of chronic diseases such as obesity, metabolic syndrome or cardiovascular diseases, being also directly related to the individual’s physical fitness. Therefore, it is necessary to measure and monitoring the levels of PA in childhood and adolescence, since it may be useful to describe their current health status and the association with physical fitness, as well as to reveal putative consequences in the future. Within the educational field, it has been shown that physical education (PE) classes are a favorable context for the creation of healthy physical-sports habits and self-determined motivation could be a key for explaining the level of PA practice. For this reason, the purpose of this research was to study the mediating role of integrated regulation (IR) on the relationship between PA and physical fitness in children and adolescents. A total of 325 students between 11 and 14 years old participated in the study. The level of PA was estimated through the specific *Physical Activity Questionnaire for Children* (PAQ-C), while motivation and IR were measured by using the *Perceived Locus of Causality scale* (PLOC-2). In addition, physical fitness was measured through the *Eurofit fitness battery of tests*, composed of three body composition measures and seven tests of different physical capacities. The physical fitness score showed no differences when genders were compared. After regression analysis, the resulting models revealed a good adjustment and correlation between PA practice and physical fitness (β = 0.173; *p* < 0.001), being established through the macro *Process* that this relationship is partially conditioned by the IR (β = 0.122; *p* = 0.03). ROC curve analysis estimated the score of 5.88 as a cut-off point to discriminate between levels of IR for students, classified as physically active or not (AUC = 0.67; *p* < 0.001). The conclusions from these main and other complementary analyses reporting complete mediations, suggest that the IR could be decisive in predicting and explaining the relationship between the practice of PA and physical fitness at these ages, highlighting its importance for a greater adherence to the practice.

## Introduction

Physical activity (PA) can be defined as any body movement produced by the action of skeletal muscles that causes an increase in energy expenditure (Rauner et al., 2013; Voss et al., 2017; Chacón-Cuberos et al., 2018a). Its practice is considered a key factor in the development, promotion and maintenance of healthier lifestyle habits at any age (Møller et al., 2009; Dumith et al., 2011; Chacón-Cuberos et al., 2018a; Castro-Sánchez et al., 2019; Zurita-Ortega et al., 2019). The physiological and psychological benefits of PA have been widely studied, highlighting the physiological ones, through the prevention and treatment of various chronic diseases such as obesity, cardiovascular disease, and metabolic syndrome (Cole et al., 2000; Webber et al., 2008; Møller et al., 2009; Ahn et al., 2011; Welk et al., 2011; Benítez-Porres et al., 2016a, b; Fang et al., 2017; Chacón-Cuberos et al., 2018a; Zurita-Ortega et al., 2019). Despite these benefits, the current scientific literature seems to have found sufficient evidence to affirm that there is a decreasing trend in PA practice worldwide (Adams, 2006; Webber et al., 2008; Owen et al., 2009; Dumith et al., 2011; Wang et al., 2016; Fang et al., 2017; Palou et al., 2019; Sierra-Díaz et al., 2019). More specifically, the World Health Organization (WHO) has identified such physical inactivity as a major global problem and has ranked it as the fourth largest risk factor for mortality worldwide (Dumith et al., 2011; Wang et al., 2016; Sierra-Díaz et al., 2019). This physical inactivity is the cause of the increase and prevalence of higher levels of obesity and overweight in the population, with sectors being particularly affected in the last decade, identified as children and adolescents (Adams, 2006; Ness et al., 2007; Webber et al., 2008; Dumith et al., 2011; Henriksson et al., 2016; Wang et al., 2016; Fang et al., 2017; Chacón-Cuberos et al., 2018b; Palou et al., 2019; Sierra-Díaz et al., 2019). In this line, the current data show worrying statistics, since a high percentage of this population are below the minimum PA practice levels pre-established by the WHO. This institution recommends at least 60 min per day of moderate physical activity (MPA) and vigorous physical activity (VPA) for the maintenance of adequate levels in order to provide health benefits at these ages (Riddoch et al., 2007; Nader et al., 2008; Webber et al., 2008; Steele et al., 2009; Dumith et al., 2011; Ekelund et al., 2011; Griffiths et al., 2013; Benítez-Porres et al., 2016a; Fang et al., 2017; Onetti-Onetti et al., 2019; Riso et al., 2019). Furthermore, scientific research in recent years has supported a focused reduction in these levels of PA practice at early ages, highlighting the fact that, as in primary education, there is a decreasing tendency for PA practice levels year by year, with an increasing inactivity trend continuing through adolescence and even adulthood (Nader et al., 2008). These tendencies put the situation in the spotlight due to the implications on the current and future health of the population (Webber et al., 2008; Collings et al., 2013; Henriksson et al., 2016; Fang et al., 2017). Therefore, the study and monitoring of PA practice levels in childhood and adolescence should be a priority, since their maintenance at these ages is considered a strong predictor of adequate PA practice levels in adult life (Møller et al., 2009; Riddoch et al., 2009; Rauner et al., 2013; Henriksson et al., 2016; Chacón-Cuberos et al., 2018b; Sierra-Díaz et al., 2019).

As a complement to the concern and importance of PA practice, current research focuses on combined studies with fitness levels (PF) (Ruiz et al., 2011; Rauner et al., 2013; Henriksson et al., 2016; Schutte et al., 2016; Wang et al., 2016; Palou et al., 2019; Riso et al., 2019; Tomkinson et al., 2019; López-Gil et al., 2020), and also including other variables such as obesity (Ness et al., 2007; Steele et al., 2009; Ekelund et al., 2011; Collings et al., 2013; Rauner et al., 2013; Benítez-Porres et al., 2016b; Henriksson et al., 2016; Fang et al., 2017; Palou et al., 2019), eating habits (Agostinis-Sobrinho et al., 2018; Ramírez-Vélez et al., 2018; López-Gil et al., 2020), physical self-concept or body image (Fernández-Bustos et al., 2019; Onetti-Onetti et al., 2019), and the motivational climate (Chacón-Cuberos et al., 2018a, b, 2019; Castro-Sánchez et al., 2019; Zurita-Ortega et al., 2019), among others.

Physical fitness is defined as the set of attributes that are achieved or possessed in relation to the ability to perform PA (Pate, 1988; López-Gil et al., 2020) or exercise with a certain skill influencing the sports performance (Schutte et al., 2016). It is considered an essential prerequisite for both, the development of daily activities without fatigue and the participation and performance of activities in free-time and leisure (Cvejic et al., 2013; Rauner et al., 2013; Schutte et al., 2016). The PF components within the health area, are cardio-respiratory, muscular and motor fitness, flexibility and body composition (Rauner et al., 2013; Kolimechkov, 2017; López-Gil et al., 2020).

Physical fitness along with PA play a key role in the health status, with the study of their interaction being a major research topic (Dollman et al., 2005; Ardoy et al., 2011; Rauner et al., 2013; Henriksson et al., 2016; Wang et al., 2016; Palou et al., 2019; Riso et al., 2019; López-Gil et al., 2020). However, this interaction can result in two different situations. For instance, a greater or lesser participation or PA practice is observed as a consequence of PF levels or the capacity to perform PA and exercise. In other words, a high level of PF could lead to a positive impulse that would generate a higher level of participation and practice of PA (Dollman et al., 2005; Schutte et al., 2016). On the other hand, scientific evidence also points out the level of PA practice as a predictor of the PF level (Dumith et al., 2011; Rauner et al., 2013; Fang et al., 2017; Palou et al., 2019), being its relationship similar to the one described above. Given higher levels of PA practice, an increase in PF levels is expected. Conversely, lower levels of PF will also be observed in situations where the levels of PA practice are low. Despite the fact that the directionality of the relationship between PF and PA does not seem to be completely clear, PA appears to be significantly conditioned by the levels of PF present in schoolchildren at this age, with aspects such as aerobic and motor fitness playing a predominant role. Therefore, low PA could be an underlying cause of the observed decrease in PF in schoolchildren (Barnett et al., 2009; Aggio et al., 2012; Fang et al., 2017). In any case, the combined association and study of PA and PF is considered a determining factor for both, follow-up and the improvement of health at all ages, especially in the children and adolescent population (Ardoy et al., 2011; Rauner et al., 2013; Henriksson et al., 2016; Fang et al., 2017; Palou et al., 2019; Riso et al., 2019).

Moreover, the habits acquired at this age will be often reproduced in adulthood, and these factors could influence and explain the acquisition of occasional healthy life-style (Chacón-Cuberos et al., 2018b; Fernández-Bustos et al., 2019; Sierra-Díaz et al., 2019). Consequently, it is necessary to gain insight studying not only the level and relationship between PA and PF, but also the reasons why the balance leans in one direction or another toward the acquisition of whether active and healthy or harmful and inactive physical sports habits. Considering that at these ages most of the day is spent in the classrooms (Ahn et al., 2011), the educational centres become the ideal location where to investigate, with the physical education (PE) playing a preponderant role (Ahn et al., 2011; Trigueros et al., 2017; Chacón-Cuberos et al., 2019; Fernández-Bustos et al., 2019) as a consequence of the environment and contents developed within this subject (Sierra-Díaz et al., 2019). PE classes are an ideal context for the creation of adherence and healthy living habits promotion associated with PA practice beyond the classroom itself (Moreno et al., 2008, 2009; Ahn et al., 2011; Dudley et al., 2011; Gillison et al., 2013; Sierra-Díaz et al., 2019). However, in order to be sure of the impact and influence of the PA, it is necessary to consider its study under the perspective of the Self-Determination Theory (SDT) (Deci and Ryan, 1985; Chacón-Cuberos et al., 2018a,b; Sierra-Díaz et al., 2019; Zurita-Ortega et al., 2019). The SDT allows to explain the reasons leading an individual to start, continue or abandon the practice of PA within the study context (Deci and Ryan, 1985; Ryan and Deci, 2000; Dudley et al., 2011; Gillison et al., 2013). The SDT is regulated by the differentiation of three types of motivation depending on the self-determination degree: intrinsic motivation (IM), extrinsic motivation (EM), and de-motivation (DE) (Deci and Ryan, 1985; Ryan and Deci, 2000; Gillison et al., 2013; Sierra-Díaz et al., 2019). IM justifies the participation and performance of activities by personal and inherent issues as a result of the enjoyment, novelty, or satisfaction involved in performing PA itself within the PE classroom. IM is the most self-determined motivation. The EM is based on the premise that external or environmental factors exist as conditioning elements in the students’ behavior and participation at this age within PE classes. Four types can be differentiated: integrated, identified, introjected, and external regulation, from highest to lowest self-determination level, respectively. Each of them is identified with different reasons and/or incentives to perform PA within the PE classroom. For integrated regulation (IR), the manifestation of a behavior would allow the integration of PA practice as another routine within those considered as mandatory for the school’s daily life (Ferriz et al., 2015; Trigueros et al., 2017). This type of regulation is identified with the last previous step of recognizing the personal value that PA practice would have in this case, and is the most self-determined within EM (Deci and Ryan, 1985; Ryan and Deci, 2000; Gillison et al., 2013). On the other hand, within the educational field for PE, the most self-determined expressions of motivation are associated with greater PA practice outside the classroom, with the scientific literature pointing directly to IR as the most determining motivation when explaining the involvement or not of schoolchildren in extracurricular PA (Wilson et al., 2006; Ferriz et al., 2015; Trigueros et al., 2017).

Finally, DE is identified as the absence of any type of interest toward the realization of physical-sporting activity at these ages, within the context of the educational environment for the PE (Ryan and Deci, 2000; Moreno et al., 2009; Dudley et al., 2011; Gillison et al., 2013; Sierra-Díaz et al., 2019). Thanks to this precept, it is possible to identify the causes originating some preferential behaviors toward the practice of PE at these ages, as well as the possible influence and relationship on the active and healthy life habits acquired for this population outside the classroom (Deci and Ryan, 1985; Moreno et al., 2008, 2009; Dudley et al., 2011; Gillison et al., 2013; Castro-Sánchez et al., 2019; Sierra-Díaz et al., 2019; Zurita-Ortega et al., 2019).

In spite of the relationships that may exist between PA, PF and motivation, few studies have analyzed these factors simultaneously outside of the university educational context. Therefore, the main objectives of this study were to analyze: (a) the relationship between the level of PA practice and the level of physical fitness in PE students, and (b) the mediating role of IR on the relationship between PA and physical fitness in schoolchildren.

## Materials and Methods

### Design and Participants

A descriptive and cross-sectional study design with non-probability-based sampling was used. The sample was selected using a sampling for convenience. A total of 325 healthy schoolchildren aged between 11 and 14 years old from five primary schools in the Region of Murcia (Spain) participated in the study. A balanced percentage distribution by gender was provided with a sample composed by male [*n* = 164 (50.5%)] and female [*n* = 161 (49.5%)] students. Age and anthropometric characteristics data, including BMI classification (Cole et al., 2000) are shown in Table 1.

**TABLE 1:** Sample descriptive data according to the gender.

	**Total**	**Boys**	**Girls**	***p***	***d***
	**(*n* = 325)**	**(*n* = 164; % = 50.46)**	**(*n* = 161; % = 49.54)**		
	**M ± SD**	**M ± SD**	**M ± SD**		
Age (years)	12.391.03	12.511.02	12.271.03	0.063	0.112
Height (cm)	1.500.08	1.490.08	1.500.08	0.131	–0.149
3rd percentile [%(n)]	16 (52)	32 (19.6)	20 (12.4)		
15th percentile [%(n)]	26.7 (87)	48 (29.4)	38 (23.6)		
Median [%(n)]	50 (162)	72 (43.6)	91 (56.5)		
85th percentile [%(n)]	6.1 (20)	10 (6.1)	10 (6.2)		
97th percentile [%(n)]	1.2 (4)	2 (1.2)	2 (1.2)		
Weight (Kg)	45.1914.27	43.5613.29	46.8615.06	0.021*	–0.168
Span (m)	1.510.10	1.510.10	1.520.10	0.298	–0.116
BMI (kg/m^2^)	19.924.82	19.394.43	20.455.15	0.068	–0.117
Normal weight [%(n)]	71.69 (233)	73.17 (120)	70.19 (113)		
Over weight [%(n)]	18.77 (61)	18.29 (30)	19.25 (31)		
Obese [%(n)]	9.54 (31)	8.54 (14)	10.56 (17)		
Physical fitness					
CRF (mL/kg/min)	39.985.38	40.945.65	39.004.92	< 0.001**	0.232
Handgrip strength (kg)^†^	19.6519.24	20.2426.58	19.045.43	0.113	–0.102
Relative Handgrip strength (kg/mass kg)^†^	0.430.11	0.430.11	0.420.11	0.234	0.133
Standing Broad Jump (m)	1.280.24	1.34.24	1.230.22	< 0.001**	0.492
Standing Broad Jump (cm)	128.2523.69	133.8523.80	122.5522.11	< 0.001**	0.492
Sit-ups (n total)	19.545.55	20.205.54	18.885.51	0.032*	0.239
Shuttle run 5 × 10 m (s)	16.301.64	15.921.66	16.691.52	< 0.001**	–0.305
Sit and reach (cm)	14.8910.70	11.559.70	18.2810.63	< 0.001**	–0.350
PFGS	0.002.35	−0.0622.37	0.0632.34	0.631	–0.053
Physical activity total score	2.970.73	3.030.77	2.910.68	0.119	0.182
Integrated regulation	5.901.11	5.901.17	5.911.06	0.756	0.019

kg, kilograms; cm, centimeters; m, meters; BMI, body mass index; mL, milliliters; min, minute; CRF, cardiorespiratory fitness; PFGS, physical fitness global score; ^†^, mean handgrip right and left; n, number; s, seconds; *p* = significance level; *d* = effect size (*d* Cohen); n, number; % percentage; **p < 0.01; *p < 0.05.

### Sample Size

The calculations to establish the sample size were performed using G^∗^Power 3.1.9.4 software. The significance level was set at α = 0.05. Accordingly, the sample size (power analysis) revealed that 306 participants would obtain a 95% power to significantly detect a correlation of *r* = 0.30 in the population (Østerås et al., 2017). In order to prevent possible dropouts or elimination of recorded data by detection of abnormal response, we decided to recruit a higher number of participants.

### Measurements and Materials

#### Physical Activity and Perceived Locus of Causality

In order to provide an estimate of the MPA to VPA levels, all students completed the international *Physical Activity Questionnaire for Children* (PAQ-C) (Crocker et al., 1997; Voss et al., 2013) validated for children aged 8–14; using the validated Spanish version by Manchola-González et al. (2017). PAQ-C is a 7-day recall composed of nine items about the frequency of physical activities at school, at home, and during leisure time (Kowalski et al., 1997). It contains nine items rated on a five-point Likert scale, where each item is rated between 1 (low PA) and 5 (high PA). Once the values were obtained for each of the items that compose the questionnaire, a final score was computed (total PA) by calculating the average value obtained in the 9 items. The PA total score allows differentiating and classifying the schoolchildren as sedentary or active according to the data obtained with the PAQ-C tool. There are several possibilities within this classification, such as the one given by Chen et al. (2008), who assigned PAQ scores ≤2 as “low activity,” >2 and ≤3 as “moderate activity” and >3 as “high activity.” There are also several studies that subdivide the sample into “active” and “low active/non-active” youth, such as Ogunleye et al. (2012), who divided youth into “active” and “low active” based on the median distribution of PAQ scores by age and sex. Along the same lines, Cervantes et al. (2017) differentiated between active schoolchildren when scores were above 3, and sedentary schoolchildren with scores below 3. Finally, Benítez-Porres et al. (2016a) established a ranking and differentiation between active and non-active schoolchildren based on a cut-off point of PAQ-C > 2.75. As a consequence, in this study the consideration and classification of the schoolchildren as active or non-active was established according to a mean score of 3 (Cervantes et al., 2017). The questionnaire shows an internal consistency value with a Cronbach’s alpha coefficient (α) of 0.83 and a good or excellent reliability value with an inter-class correlation coefficient (ICC) of >0.73 and a 95% confidence interval.

Similarly, students performed the *Perceived Locus of Causality Scale* (PLOC-2) (Ferriz et al., 2015). Through this questionnaire, the scores obtained by the schoolchildren for the different types of motivation established by the SDT within the PE classes were recorded. The questionnaire arises as a consequence of the fusion of the Perceived Locus Causality Scale (PLOC) (Moreno et al., 2009) and the four items elaborated by Wilson et al. (2006) to measure IR. The scale is headed at the beginning by the phrase “I participate in PE classes….” It is composed of 6 factors and a total of 24 items distributed by four for each factor. The components of the scale are: IM (e.g., “because PE is fun”), IR (e.g., “because it’s in line with my way of life”), identified regulation (e.g., “because I want to learn sports skills”), introjected regulation (e.g., “because I would feel bad about myself if I didn’t”), external regulation (e.g., “because that’s what I’m supposed to do”), and demotivation (e.g., “I really feel like I’m wasting my time in PE classes”). The instrument uses a Likert scale ranging from 1 to 7 where 1 is identified as disagreeing strongly and 7 as agreeing strongly. The reliability of the scale measured through Cronbach’s alpha (α), obtained the following results by factors: α = 0.84 (IM); α = 0.93 (IR); α = 0.84 (identified regulation); α = 0.69 (introjected regulation); α = 0.69 (for external regulation) and α = 0.82 (demotivation). Confirmatory factor analysis showed the following adjustment rates: χ2 (235, *N* = 858) = 1147.31, *p* < 0.001, χ2/gl = 4.88, CFI = 0.92; IFI = 0.92, TLI = 0.91, RMSEA = 0.065 (IC 90% = 0.063–0.071) and SRMR = 0.065. Finally, factorial weights ranged from 0.47 to 0.90.

#### Physical Fitness Assessment

Several studies analyzing the PF as predictor of PA (Jaakkola et al., 2015) or the use of muscle strength field-based test to identify risk in several diseases among adolescents (Castro-Piñero et al., 2019) used a battery of PF assessment in order to obtain scientific support and strong correlations between key factors related to the health status in young population. The level of physical fitness and the different components (core strength, lower and upper body strength, agility, balance, flexibility, and endurance) were assessed using the *Eurofit Physical Fitness Test Battery* (Gulías-González et al., 2014; Purohit et al., 2016). The protocols established by the guidelines for this test battery and the American College of Sports Medicine [ACSM], 2010 were followed to guarantee the safety of the participants. In addition, to ensure the successful performance in the Test Battery, all students were informed about the protocol to perform in the experimental sessions. A usual warm-up (mostly running and dynamic stretching exercises) during 7–8 min was carried out before physical testing. The better result of two attempts per hand (*handgrip test* and *5 × 10 m shuttle run*) and three attempts for *standing broad jump* and *sit and reach test* were recorded, while only one successful attempt was allowed for the *sit-ups test* and *20 m Shuttle Run*. All tests were conducted indoors (school gym) wearing comfortable sporting attire. Jumping and running tests were carried out on wooden non-slippery floor. The recovery time between attempts was set at 2 min, except for the 5 × 10 m shuttle run test, with a complete recovery between attempts. The raw scores obtained in each physical fitness test were transformed into standardized scores (z-scores) for the entire sample. Then, an overall physical fitness general score (PFGS) was calculated. The overall PFGS score was obtained by calculating the average of the z-scores obtained in each of the PF tests (Jaakkola et al., 2015; Østerås et al., 2017; Castro-Piñero et al., 2019).

##### Body composition characteristics measurement

The anthropometric measurements made for the determination of weight, height and span followed the standards established by the International Society for the Advancement of Kinanthropometry (ISAK) (Esparza-Ros et al., 2019). Body weight (kg), body mass index (BMI) and % body-fat were measured without shoes using a Bioelectrical impedance analysis device (BIA) (Tanita BC-545N–Body composition monitor to measure per segment) to the nearest 0.1 kg. The Height (cm) was measured using a stadiometer (Tanita HR001 Leicester portable height rod) to the nearest 0.1 cm. The span was measured (in meters) using an anthropometric wall tape (Lufkin L1025B) and metal standoffs. The requirements and clothing for these measures were established beforehand through the information provided in the dossier given to the PE teacher, as well as in the information letter provided to parents or legal tutors and the students themselves. It was established that students should come with comfortable sportswear (short-sleeved shirt and shorts), and their height and weight must be taken barefoot. The established protocol was performed in order to ensure an adequate data collecting process during all the tests.

##### Cardiorespiratory fitness (CRF)

A maximum incremental field test *(20-m endurance shuttle run test)* was performed in order to estimate the maximum volume of oxygen (MOC) consumed. The test involves running between two lines separated by 20 m while following the rhythm of acoustic signals. The initial speed was set at 8.5 km/h increasing by 0.5 km/h per minute (maximum of 18.0 km/h at 20th min). Subjects were instructed to run maintaining the pace, follow the straight running line and turn properly after completing each lap. Verbal encourage was provided to ensure a full-effort test. When the subject did not reach the end line twice following the acoustic signal or stopped completely due to the fatigue, the test was finished. Léger’s et al. (1988) equations were used in order to transform the states to relative MOC values (López-Gil et al., 2020).

##### Upper and lower body strength

Upper body muscular strength was assessed using the *handgrip strength* by a hand dynamometer with adjustable grip (TKK 5101 Grip-D, Takei^®^, Tokyo EH101) recording the scores in kilograms (Kg). The reported precision of the dynamometer was 0.1 kg. A short demonstration and verbal signal were provided during the test and the grip was adjusted to the hand size. The hand span was measured in both hands from the tip of the thumb to the tip of the small finger with the hand wide opened as much as possible. Therefore, the optimal grip span was selected (boys and girls) following the recommendations by España-Romero et al. (2008). The minimum time required was set at 2 s (maximum at 5 s) and two attempts per hand were allowed. The analysis was performed using the averaged best scores reached by each hand. A normalization was calculated and expressed as kg/mass kg (Ruiz et al., 2011). The *sit-up test* measures abdominal muscles function as number of sit-ups completed from lying position (knees bent at a 90°) in 30 s. The test was performed on floor mate and Casio handheld stopwatches (HS-80TW-1EF) were used for recording the time. Moreover, the *standing broad jump test* has been successfully used for measuring the lower limb explosive strength. Participants jumped horizontally from the starting line to achieve maximum distance (in cm). The test was performed three times and the best score was kept for further analysis. The test was performed in an indoor school gym in order to avoid falls caused by slipping and a classic anthropometric tape (Lufkin L1025B) of 5 m length and 3.4 cm wide was used for distance measuring.

##### Motor fitness and flexibility

Speed and agility were evaluated by the *5 × 10 m Shuttle test*. The participants were required to complete five repetitions of 10 m distance at the maximum possible speed between two lines placed at 5 m each other. The results were measured in seconds using Casio handheld stopwatches (HS-3V-1). Measuring tape and marker cones were used to perform the test. The best score of two attempts was used for further analysis (López-Gil et al., 2020). The *sit and reach* test was performed following specific instructions. Lower body flexibility was assessed while attempting to reach forward as far as possible keeping knees straight in a sitting position. Sit and reach box was used to record the distance reached by the hand in cm.

### Procedures

#### Informed Consent and Previous Information

The following protocol was established to ensure similar data collection in all centres and throughout the whole process. Firstly, the schools were contacted, and a meeting with the directors was requested to inform them about the research purpose and to request their participation. After the acceptance, a meeting with the PE teachers was held. At this meeting, they were provided with all the necessary documentation and all doubts regarding the measurement protocol to be followed, instruments and tests to be carried out, were explained. In addition, the facilities and spaces available within the educational centre were visited and organized. Secondly, an adequate and similar schedule about the days and time slots was agreed. Prior to the intervention, all students and their parents or legal tutors were informed about the study characteristics as well as the possible benefits and potential risks. Subsequently, a consent form to voluntarily participate in the study was fulfilled and signed.

The study was conducted in accordance with the ethical principles of the Helsinki declaration for human research (World Medical and Association, 2013) and was approved by the institutional review board of the Catholic University San Antonio of Murcia (Code: CE031802).

Researchers visited the school in two different days (first and second phase), always at the same time of the day (10:30 am–02:30 pm), separate by at least 48 h and no more than 72 h and controlling the environmental conditions (space, temperature, and humidity). Thus, all tests were performed at the same location and time as well as with similar humidity (30–40%) and temperature (20–24°C) conditions.

#### Measures

*Physical Activity Questionnaire for Children* and PLOC-2 (approximately 30 min) were performed during the first phase (First session) by the participants. An investigator provided the questionnaires to the participants and informed them how to fill them in. In order to carry out the different questionnaires effectively, several research assistants explained the questionnaires and test protocols solving possible questions during the process. Once the questionnaires were completed, the body composition measurements and *handgrip strength test* were registered following the protocols detailed previously. In order to carry out the different tests, the participants were divided into two equal groups, making rotation (station 1: weight, height and span; and station 2: *handgrip strength test* – dynamometry). Each group was guided by a researcher who was responsible for explaining and controlling the performance of each test. The first phase was completed in 1 h.

**FIGURE 1:**
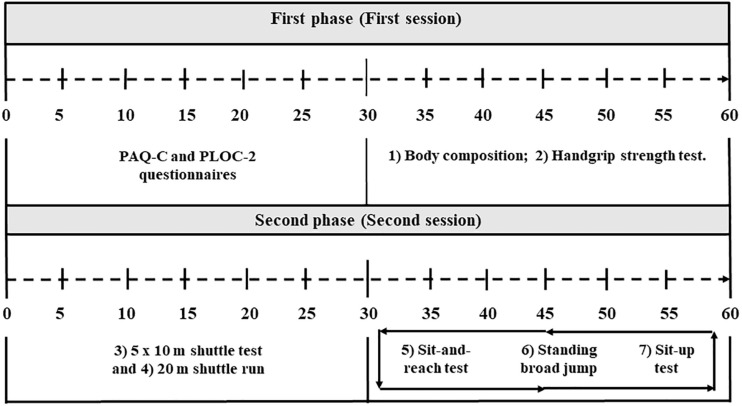
Schematic representation of first phase (see text for full description).

In the second phase (second session), students performed the *5 × 10 m shuttle test*, *20 m shuttle run* (first post), *the sit-and-reach test, standing broad jump* and *sit-up test* (second post). The participants kept the division in equal groups, making rotations to conduct the tests correctly. The Figure 1 shows the complete data collection process during all the tests in both phases.

### Statistical Analysis

Means (M), standard deviations (SD), percentages (%) and frequencies were calculated using descriptive statistics. Data normality and homogeneity (Kolmogorov–Smirnov and Levene tests) were conducted prior further analysis. Data variables with different units and arithmetic scales were transformed on standardized scores (Z score). Subsequently, when differences between groups were observed, an independent sample *t*-test, Mann Whitney *U* test or Pearson’s chi-squared test (χ^2^) were used, depending on the assumption of normality. A *post-hoc* comparison for the 2xn tables was applied (statistic contingency coefficient); including statistic value and *p*-value. The maximum expected value was 0.703; showing a small association *r* < 0.3; the *r-*value of the moderate association was between 0.3 and 0.5 and the high association *r* > 0.5. Effect size was calculated by Cohen’s *d* [0.2 (small); 0.5 (medium) and >0.8 (large) effect]. In addition, multiple regression analysis was performed to determinate relationship between variables. In all cases, data independence, absence of collinearity, normality and homoscedasticity were verified. The existence of relationship between these variables was measured by Rho (ρ) of Spearman and Pearson correlation coefficients, accordingly.

In order to assess whether the association between main variables, a multiple moderated mediation analysis was performed. The mediation analysis was performed using the PROCESS macro from SPSS (SPSS Inc., Chicago, IL, United States). The resampling procedure of 10,000 bootstrap samples was used for the non-parametric sample (Preacher and Hayes, 2008), while the classical method of stepwise regression by Baron and Kenny (1986) was used for the parametric sample. Statistical significance of mediation effect was examined by test of Sobel (1982). When the *z* value was higher than 1.96 and *p* value was lower than 0.05 a mediating effect is admitted (Sobel, 1982; Preacher and Hayes, 2008).

Finally, a ROC (Receiver Operating Characteristics) curve analysis was performed to determinate the precise cut-off points of IR variable in the determination and classification of the study participants according to their classification (physically active or not). The classification accuracy for each set of cut-off points was evaluated by calculating weighted statistics, sensitivity, specificity and area under the receiver operating characteristic curve (AUC). An area of one represents a perfect rating, while an area of 0.5 represents an absence of rating accuracy. ROC-AUC values of >0.90 are considered excellent, 0.80–0.89 good, 0.70–0.79 fair, and <0.70 poor (Metz, 1978). Data analysis was performed using the software SPSS (IBM Corp., Armonk, NY, United States) for Windows, Version 24.0, as well as MedCalc 14.12.0 (Mariakerke, Belgium). The statistical significance level was set at 0.05 for all statistical comparisons.

## Results

The general and individual scores for each PFGS tests, as well as the PA level for both, overall level and according to gender are reported in Table 1. Concerning to the anthropometric characteristics, significant gender differences were only observed for the weight variable (*p* = 0.021, *d = −*0.168). Regarding the scores in the different PF tests according to gender, statistically significant differences were found for CRF (*p* < 0.001, *d = −*0.232), sit-ups (*p* = 0.032, *d* = 0.239), length jump (*p* < 0.001, *d* = 0.492), 5 × 10 m shuttle run test (*p* < 0.001, *d = −*0.305) and flexibility (*p* < 0.001, *d = −*0.348), finding higher values in girls compared to boys only for the last test.

**FIGURE 2:**
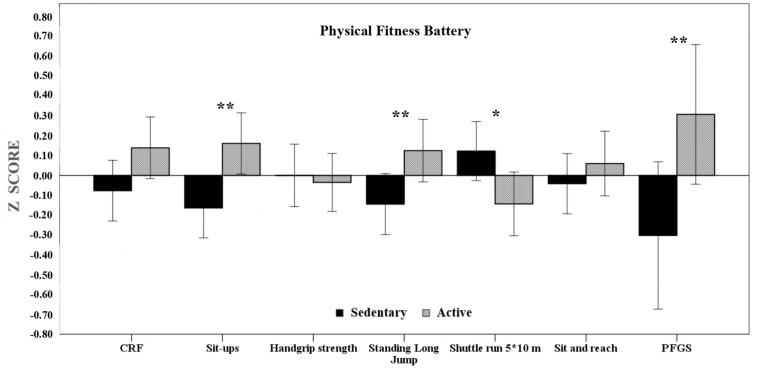
Physical Fitness Batery [Z Scores] depending on Physical Activity Total Score. Data as shown as mean. **p < 0.01; *p < 0.05. Error bars indicate 95% confidence intervals.

Likewise, the anthropometric characteristics, as well as the individual scores for each PF tests according to the PA total score are shown in Table 2. For anthropometric characteristics, only differences in weight or BMI were obtained. In addition, we observed significant differences for the standing broad jump, sit-ups and shuttle run 5 × 10 m tests in the individual tests of the PF battery. According to the classification as sedentary or active schoolchildren, these differences were significant in the PFGS. Finally, there were also differences in the score for the IR. Specifically, the differences in individual PF tests and in the PFGS are displayed in Figure 2.

**TABLE 2:** Sample descriptive data according to the Physical Activity Total Score.

**Physical Activity Total Score (PAQ-C)**						
	**Sedentary**		**Active**		***p***	***d***
	**M**	**SD**	**M**	**SD**		
Height (cm)	1.50	0.09	1.49	0.08	0.178	0.151
Weight (Kg)	46.56	14.62	43.39	12.88	0.043*	0.136
Span (m)	1.52	0.10	1.51	0.09	0.174	0.1000
BMI (kg/m^2^)	20.31	4.74	19.36	4.55	0.048*	0.1333
**Physical fitness**						
CRF (mL/kg/min)	39.42	5.58	40.60	5.13	0.061	–0.121
Handgrip strength (kg)^†^	18.57	4.74	18.28	3.96	0.807	0.03
Relative Handgrip strength (kg/mass kg)^†^	0.45	4.74	0.44	3.96	0.641	0.03
Standing Long Jump (m)	1.25	0.24	1.32	0.23	0.007**	–0.301
Sit-ups (n total)	18.55	5.55	20.62	5.35	0.001**	–0.379
Shuttel run 5 × 10 m (s)	16.52	1.62	16.06	1.63	0.021*	0.149
Sit and reach (cm)	14.32	10.80	0.53	15.54	0.370	–0.059
PFGS	–0.31	2.45	0.34	2.20	0.013*	–0.280
Integrated regulation	5.59	1.23	6.27	0.82	< 0.001**	0.336

kg, kilograms; cm, centimeters; m, meters; BMI, body mass index; mL, milliliters; min, minute; CRF, cardiorespiratory fitness; PFGS, physical fitness global score; ^†^, mean handgrip right and left; n, number; s, seconds, *p* = significance level; *d* = effect size (*d* Cohen); **p < 0.01; *p < 0.05.

The different bivariate correlations according to the obtained score for the PF score index in relation to the IR and PA variables are presented in Table 3. Positive and statistically significant correlation was found between the PF score index and the PA level (*R* = 0.174, *p* = 0.001), the PF score index and IR (*R* = 0.138, *p* = 0.003) and between the predictor variables of PA level and IR (*R* = 0.324, *p* < 0.001).

**TABLE 3:** Bivariate correlations between Physical Fitness Index Score and the related variables of the study.

	**1**	**2**	**3**
1. Physical Fitness Index Score	1		
2. Physical Activity Total Score	0.174**	1	
3. Integrated Regulation	0.138**	0.324**	1

**p < 0.01.

**FIGURE 3:**
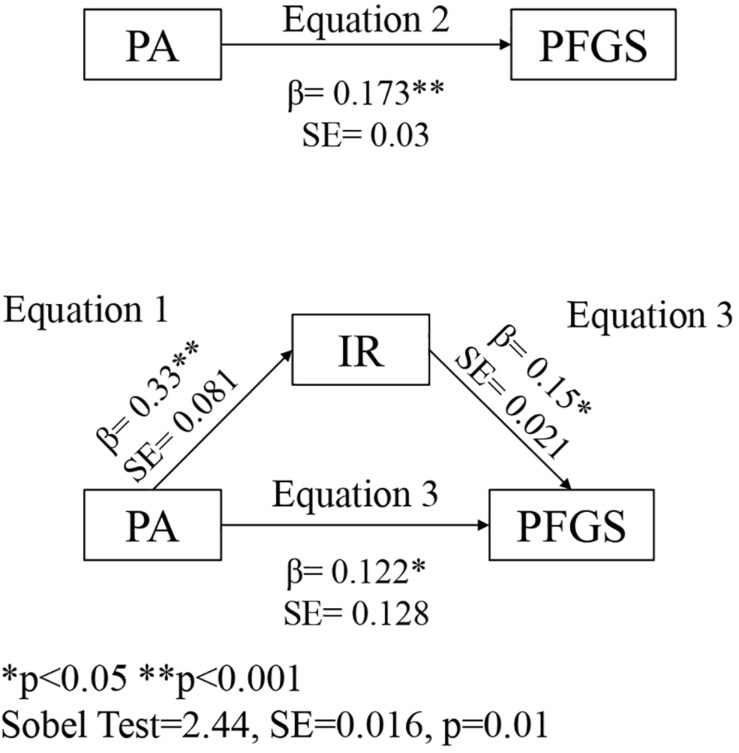
Mediation of Physical Activity (PA) and Physical Fitness Global Score (PFGS) by Integrated Regulation (IR).

The results (Figure 3) revealed that the PFGS was associated with the practice of PA (*R*^2^ = 0.03, *F* = 9.85, β = 0.173, SE = 0.03, *t* = 3.13, *p* = 0.001, 95% CI = 0.035 to 0.150) and with IR (*R*^2^ = 0.051, *F* = 8.55, β = 0.15, SE = 0.021, *t* = 2.656, *p* < 0.008, 95% CI = 0.014 to 0.095). In addition, the IR was associated with the practice of PA (*R*^2^ = 0.109, *F* = 39.13, β = 0.33, SE = 0.081, *t* = 6.25, *p* < 0.001, 95% CI = 0.347 to 0.665).

The mediating effect of IR on the association between PA practice and PFGS was also analyzed. After including IR in the equation, the association between PA practice and PFGS remained significant, but its effect was reduced (*R*^2^ = 0.051, *F* = 8.55, β = 0.122, SE = 0.128, *t* = 2.11, *p* = 0.03, 95% CI = 0.004 to 0.128), suggesting that IR is a mediating variable with a partial effect. The analysis of mediation revealed both, a significant direct and indirect effect with a significant Sobel test value (*z* = 2.44 ± 0.01, *p* = 0.01).

Area under the curve and the scores for the PAQ-C equivalent to the coordinates with the highest sum of sensitivity and specificity are shown in Tables 4, 5. The AUC of the PAQ-C score for all cases, including male and female, was significant (*p* < 0.05) and moderate discriminator was observed only for male gender (AUC > 0.7) when comparing active and non-active children, while for all case studies and female gender alone it was a weak discriminator (AUC < 0.7).

**TABLE 4:** Area under the ROC curve of PAQ-C score based on integrated regulation (IR).

**IR**	**PAQ-C score**		
	**Total (*n* = 325)**	**Boys (*n* = 164)**	**Girls (*n* = 161)**
	**Active**	**Active**	**Active**
AUC	0.668	0.714	0.611
SE	0.030	0.041	0.045
95% CI	0.614 to 0.719	0.638 to 0.738	0.530 to 0.687
*p*	<0.001**	<0.001**	0.014*
Youden index	0.281	0.371	0.219

AUC, area under the curve; SE, standard error; CI, confidence interval; *p*, significance level; **p < 0.01; *p < 0.05.

**TABLE 5:** PAQ-C score cut-off points and sensitivity, specificity, likelihood ratios, and predictive values, based on integrated regulation (IR).

	**Cut point**	**Sensitivity**	**95% CI**	**Specificity**	**95% CI**	**+LR**	**95% CI**	**−LR**	**95% CI**	**+PV**	**95% CI**	**−PV**	**95% CI**
Total	>5.75	75.17	67.4–81.9	52.91	45.2–60.5	1.6	1.3–1.9	0.47	0.3–0.6	58	53.5–62.4	71.1	64.3–77.1
Boys	>6.25	64.63	53.3–74.9	72.5	61.4–81.9	2.35	1.6–3.5	0.49	0.4–0.7	70.7	62.0–78.1	66.7	59.2–73.4
Girls	>5.75	67.74	54.7–79.1	53.33	42.5–63.9	1.45	1.1–1.9	0.6	0.4–0.9	50	38.9–61.1	70.6	58.3–81.0

*CI, confidence interval; LR, positive (+) and negative (−) likelihood ratios; PV, positive (+) and negative (−) predictive values.*

The IR score cut-off from the ROC analysis was 5.88 for the whole group and one subgroup (girls), while for boys the cut-off point was 6.37 when discriminating between active and non-active children.

**FIGURE 4:**
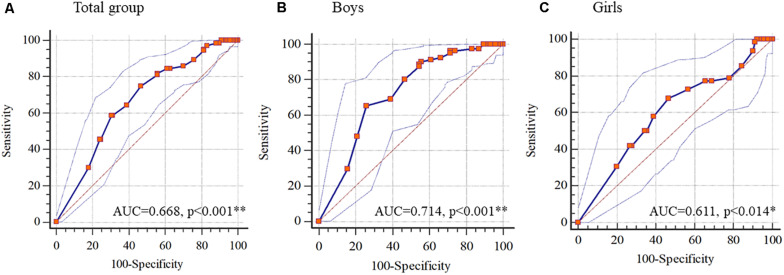
Receiver-operator curves for the Integrated Regulation (IR) to identify PAQ-C’s score of three group (**A**, Total group; **B**, Boys; and **C**, Girls). Data as shown as mean. **p < 0.01; *p < 0.05.

Sensitivity was moderate for the different groups when considering a complete group (75.15%), or separated by gender (64.63% and 67.74% for boys and girls, respectively). However, for specificity, only one factor (boys) had a moderate-high score (75.5%), while for the whole group or girls alone showed moderate values (52.91% and 53.33%, respectively). Finally, the ability of the male gender to determine the differentiation between active or non-active was higher than the ability of the IR score for female gender and overall, as evidenced by the scores for positive and negative probability ratios on this factor compared to the others (girls alone and entire group). The same applies to positive and negative predictive values. ROC curves are shown in Figure 4.

## Discussion

In the present study we describe, compare and analyze the mediating role of IR on the relationship between the PA and PF level in school children between 11 and 14 years old. The results confirmed a positive association between PA and PF variables. In addition, the data allowed us to corroborate the IR role as a partial mediator of the established relationship between both variables. In addition, we established a cut-off point to discriminate between IR levels according to the classification (physically active or not) based on the PAQ-C scores by ROC curves analysis.

Firstly, concerning the anthropometric measures, significant differences when compared the schoolchildren gender and weight were observed (slightly higher +7.04% in girls). However, there were no significant differences in the BMI or any of the other anthropometric measures. These results are consistent with those previously reported by other studies (Jaakkola et al., 2015; Fang et al., 2017; Fernández-Bustos et al., 2019; López-Gil et al., 2020). Conversely, no significant differences were observed between genders when comparing PA levels, which is not in accordance with previous research studies where evidences of higher PA practice levels for boys were reported (Owen et al., 2009; Riddoch et al., 2009; Collings et al., 2013; Griffiths et al., 2013; Voss et al., 2017). However, several studies pointed out that such differences in the PA level could be due to significant differences in BMIs, as well as the results in measures of adiposity allowing for the identification and classification of schoolchildren with or without problems of hypertension or obesity (Riddoch et al., 2007, 2009; Owen et al., 2009; Ekelund et al., 2011; Niederer et al., 2012; Collings et al., 2013; Rauner et al., 2013; Jaakkola et al., 2015; Raistenskis et al., 2016; Wang et al., 2016; Fang et al., 2017; Fernández-Bustos et al., 2019). More precisely, those studies state that children who are obese or overweighted have a higher tendency to be physically less active (Ness et al., 2007; Rauner et al., 2013; Wang et al., 2016).

Our findings did not reveal any significant difference in the BMI by gender, which could justify the lack of differences in the PA levels in our sample. Furthermore, even if it seems evident that lower BMI levels could be associated with higher PA, the desire to lose weight in female students could be the trigger for inducing a more active behavior and, therefore, increased PA practice levels (Fernández-Bustos et al., 2019), explaining the lack of differences found in this study. The obtained scores in our research for PA practice levels are very similar to those found in previous research [e.g., PA total score = 2.97, PA score girls = 2.91, PA score boys = 3.03 in our study versus PA score total = 3.09, PA score girls = 3.11, PA score boys = 3.07 (Benítez-Porres et al., 2016b) or PA score total = 2.6 (Voss et al., 2017) or PA score girls = 2.7 and PA score boys = 2.9 (Voss et al., 2013)].

Moreover, significant differences were obtained when comparing the results for the different PF tests by gender. Specifically, boys achieved higher scores on the CRF, standing broad jump, sit-ups and 5 × 10 m shuttle run tests, while girls scored better on the sit and reach test. Although our findings are consistent to other studies (Schutte et al., 2016; Fang et al., 2017; Castro-Piñero et al., 2019; Lang et al., 2019). The interpretation of the score results should be carefully considered due to the lack of significant differences in the PA levels. Several scientific evidences highlighted an association between both factors, when higher PA practice levels are registered, an increase in PF levels is expected (Dollman et al., 2005; Dumith et al., 2011; Rauner et al., 2013; Schutte et al., 2016; Palou et al., 2019; Riso et al., 2019). The most plausible explanation for these differences by gender may be due to aspects of evolutive growth, which would allow boys to perform physical tests in a different way (Fang et al., 2017). Nevertheless, when the PFGS is considered, the gender difference was not observed, suggesting that our findings are in line with those found in the literature mentioned above. Concerning the individual results, the CRF test scores for both genders showed similarities with those identified as healthy indexes (35 and 42 ml /kg /m for girls and boys, respectively) in other studies (Lang et al., 2019). The results of the scores for lower and upper body muscle strength (mean scores) are similar to those obtained by Castro-Piñero et al. (2019) and by Segura-Jiménez et al. (2016), but there were no significant differences by gender in terms of upper limb strength levels, probably due to the different tools or the measurement period during the academic year used in each study. For PF, cardiorespiratory and muscular fitness are an important health marker associated with the risk of metabolic syndrome (Webber et al., 2008; Møller et al., 2009; Ahn et al., 2011; Welk et al., 2011; Rauner et al., 2013; Benítez-Porres et al., 2016a, b; Fang et al., 2017; Chacón-Cuberos et al., 2018b; Castro-Piñero et al., 2019; Lang et al., 2019; Zurita-Ortega et al., 2019) and, therefore, both measures and results are relevant for our research.

Taking into account the relationship between PA and PF, our results reveal both, a positive association between both factors and significant differences in the scores for the individual PF tests and the PFGS according to the classification and identification of the schoolchildren as physically active or not. Specifically, our results support the evidence that at any age, being physically active is synonymous of less difficulty and effort in facing physical tasks (Jaakkola et al., 2015). This could explain the increase in PF performance for physically active schoolchildren at both, at general and individual level (Rauner et al., 2013). Contrarily, lower levels of PF were observed in inverse situations (classification of schoolchildren as sedentary) (Dollman et al., 2005; Dumith et al., 2011; Rauner et al., 2013; Schutte et al., 2016; Wang et al., 2016; Palou et al., 2019; Riso et al., 2019). Regarding the FP test data at an individual level, there is a slightly positive trend identified with a better CRF score for physically active school children. But conversely to the results of previous studies, no statistically significant differences were observed (Ardoy et al., 2011; Cvejic et al., 2013; Rauner et al., 2013; Segura-Jiménez et al., 2016; Fang et al., 2017; Kolimechkov, 2017; Castro-Piñero et al., 2019).

The differing results could be explained by the fact that is that practice intensity has not been considered in the current study when measuring PA levels. In addition, there is some controversy about using a self-reported questionnaire to measure and assess PA because it is considered an indirect and subjective measure at these ages that may lead to an overestimation of PA practice levels (Palou et al., 2019; Riso et al., 2019). There are several investigations supporting the reliability and validity of this PAQ-C instrument for collecting this information (Biddle et al., 2011; Benítez-Porres et al., 2016a; Wang et al., 2016; Voss et al., 2017; Hidding et al., 2018), and in addition, the Spanish adaptation obtained a high test-retest reliability (ICC = 0.96) and a Cronbach’s Alpha coefficient of α = 0.76, which allows us to state a satisfactory internal consistency (Benítez-Porres et al., 2016b). Riddoch et al. (2009) made two very important findings that could explain the discrepancies found in this research compared to the previous scientific literature. Thus, the reality of PA practice at these ages differs from adults or adolescents. A high percentage of schoolchildren do not comply with the minimum recommendations for MPA and VPA practice preestablished by the WHO. Furthermore, PA is rarely performed during prolonged periods and intensities providing a good cardiorespiratory condition, since their normal activity rhythms are mostly characterized by short periods of activity.

On the other hand, schoolchildren classified with a physically active profile obtained higher scores for FP tests related to agility (Shuttle run 5 × 10 m), abdominal strength-resistance (abdominals), lower body strength (Standing Broad Jump) and flexibility (Sit and reach), obtaining an overall index (PFGS). These data show clear significant differences depending on whether they are identified as physically active or not.

In summary, our research findings are in line with scientific evidence showing differences in PF level according to PA levels (Dollman et al., 2005; Ardoy et al., 2011; Dumith et al., 2011; Cvejic et al., 2013; Rauner et al., 2013; Schutte et al., 2016; Segura-Jiménez et al., 2016; Wang et al., 2016; Fang et al., 2017; Kolimechkov, 2017; Castro-Piñero et al., 2019; Palou et al., 2019; Riso et al., 2019). The differences observed between the PFGS score in this investigation and other studies could be due to the lack of unification (aspects to be considered in the PF assessment) since each study measures different tests and/or particular aspects (in some studies only aerobic fitness, muscle strength or both are measured). Ardoy et al. (2011) highlights the promotion of other PF components along with those mentioned above, such as flexibility and agility, since they are directly involved in improving PF. Finally, the significant differences found when comparing active and non-active school children for weight and BMI reinforce our findings and statements regarding the relationship between all these elements (Ness et al., 2007; Collings et al., 2013; Rauner et al., 2013; Henriksson et al., 2016; Palou et al., 2019).

At these ages, children spend a considerable amount of time in educational institutions (Ahn et al., 2011), where the PE subject could play an important role in generating and promoting healthy lifestyles beyond the classroom itself (Moreno et al., 2008, 2009; Ahn et al., 2011; Dudley et al., 2011; Gillison et al., 2013; Fernández-Bustos et al., 2019; Sierra-Díaz et al., 2019). Through the SDT it is possible to explain the impact and influence that PE has on schoolchildren. Accordingly, our results showed significant differences in the IR score when classifying the schoolchildren as physically active or not. Moreover, when the influence of IR on the relationship between PA and PF was tested, the IR partially mediated that relationship.

Integrated regulation is the first level within the ME, which is based on the premise that external or environmental factors exist as conditioning factors to explain behavior and participation at these ages in any activity (Ryan and Deci, 2000; Moreno et al., 2009; Dudley et al., 2011; Gillison et al., 2013; Sierra-Díaz et al., 2019). Within the classroom context, one of the main purposes for the PE teacher is to achieve a better knowledge and awareness among students about the benefits of the PA practice and adequate PF levels have for their current and future health (Moreno et al., 2008, 2009; Dudley et al., 2011; Gillison et al., 2013; Chacón-Cuberos et al., 2018b; Castro-Sánchez et al., 2019; Fernández-Bustos et al., 2019; Sierra-Díaz et al., 2019). In our research, the differences between physically active and non-active schoolchildren could be related to the identification and internalization of the benefits of the PA practice for health improvement.

Additionally, the higher the student’s PF, the higher their motor skills are likely to be, which will provide feedback on this pattern of active and healthy behavior, due to the optimal experience in the motor learning and technical skills acquired at PE lessons (Sierra-Díaz et al., 2019). Moreover, if we compare physically active and non-active students, the first ones will be able to overcome the challenges with less difficulty and effort together with higher guarantees of success. This is a consequence of a higher PF level (Dollman et al., 2005; Dumith et al., 2011; Rauner et al., 2013; Schutte et al., 2016; Wang et al., 2016; Palou et al., 2019; Riso et al., 2019), which will give them greater enjoyment and satisfaction in practice (Dudley et al., 2011; Gillison et al., 2013; Chacón-Cuberos et al., 2019; Sierra-Díaz et al., 2019).

The major finding of this study was that IR could be used to discriminate between physically active or non-active schoolchildren both, on an overall assessment and differentiating by gender. The established cut-off points were 5.88 (total group and female subgroup alone) and 6.37 for boys. As far as we know, this is the first study defining these cut-off points based on IR. The AUC value (general group and girls) is close to 0.70, which could invalidate the results of the diagnostic test at the clinical level, given the potential repercussions of an erroneous classification related to the presence or absence of disease. However, both tests presented acceptable levels of specificity for such discrimination and classification (Metz, 1978). Additionally, the PLOC-2 measuring instrument that allows IR recording is not a clinical diagnostic test, and also, comparatively low or similar AUC values are continuously published within this context (Benítez-Porres et al., 2016a; Voss et al., 2017). Therefore, our results could be considered as suitable as a test for differentiating levels of IR according to this classification for schoolchildren.

Finally, even though the PAQ-C questionnaire has demonstrated its validity for recording PA levels (Biddle et al., 2011; Benítez-Porres et al., 2016a; Wang et al., 2016; Voss et al., 2017; Hidding et al., 2018), the results could be influenced by the measurement instrument, being a possible limitation of this study. For this reason, it would be interesting to support the results already obtained by using better measuring instruments for a direct quantification of the PA levels. Further research is needed in order to corroborate the main findings.

## Conclusion

To our knowledge, this is the first study that considers the mediating role of IR on the levels of PA and PF practice in school children. The findings of this novel study provide new information about the role of IR in PE classes as a supporting factor for the acquisition of not only healthy and active habits at these ages, but also for the improvement of PF levels. In addition, our findings establish a difference in the IR scores obtained by the schoolchildren according to their classification as physically active or not active, allowing us to establish cut-off points by ROC analysis. This analysis differentiates both the group of schoolchildren at a general level and by gender when classifying them according to the levels of PA practice. These cut-off points could be a useful and inexpensive way to assess the school population, in order to implement intervention strategies, especially in terms of structuring the content to be taught in the classroom. The aim is to improve motivation toward the teaching-learning process and, thus, increase the levels of PA and, consequently, improve the levels of PF.

## Data Availability Statement

The authors confirm that the data supporting the findings of this study are available within the article/Supplementary Material.

## Ethics Statement

The studies involving human participants were reviewed and approved by the University Ethics Committee of the Catholic University of Murcia (UCAM) reviewed and approved the research in accordance with the principles set out in the Declaration of Helsinki (Code: CE031802). Written informed consent to participate in this study was provided by the participants’ legal guardian/next of kin. Written, informed consent was obtained from the individuals’ legal guardian/next of kin for the publication of any potentially identifiable images or data included in this article.

## Author Contributions

GG-G and NG-G conceptualized and designed the study and carried out the statistical analysis. FG-F, LM-A, and PM-P recruited the subjects. GG-G, NG-G, and AE-G collected the data. AE-G and FG-F organized the database. GG-G, NG-G, AE-G, PM-P, FG-F, and LM-A wrote the first manuscript draft. GG-G and LM-A developed the final manuscript draft, the English proofreading, and reviewed and edited the final version of the manuscript. All authors contributed to the manuscript revision and approved the definitive manuscript.

## Conflict of Interest

The authors declare that the research was conducted in the absence of any commercial or financial relationships that could be construed as a potential conflict of interest.
